# Diagnosis and management of tuberculosis in children

**DOI:** 10.36416/1806-3756/e20250045

**Published:** 2025-03-18

**Authors:** Maria Fernanda Gonçalves Meirelles Fernandes, Gabriela de Azevedo Bastian de Souza, Júlia Giffoni Krey, Leonardo Araújo Pinto, Maria de Fátima B. Pombo Sant’Anna, Clemax Couto Sant’Anna

**Affiliations:** 1. Grupo de Pesquisa em Epidemiologia e Genética das Doenças Respiratórias da Infância, Pontifícia Universidade Católica do Rio Grande do Sul - PUCRS - Porto Alegre (RS) Brasil.; 2. Programa de Pós-Graduação em Medicina - Pediatria, Escola de Medicina, Pontifícia Universidade Católica do Rio Grande do Sul - PUCRS - Porto Alegre (RS) Brasil.; 3. Programa de Pós-Graduação em Saúde Materno-Infantil - PPGSMI - Universidade Federal do Rio de Janeiro - UFRJ - Rio de Janeiro (RJ) Brasil.

## INTRODUCTION AND EPIDEMIOLOGY

Tuberculosis is a communicable disease caused by *Mycobacterium tuberculosis*.^1^ Although preventive measures and effective treatment are available, tuberculosis remains a major cause of morbidity and mortality worldwide, particularly in resource-limited settings, exacerbating social inequality.^1^ Transmission occurs through the respiratory tract, with the inhalation of airborne particles produced by coughing, speaking, or sneezing from individuals with active pulmonary or laryngeal tuberculosis.^2^

Children and young adolescents account for approximately 11% of all tuberculosis cases globally, with an estimated 1.1 million children developing the disease each year, nearly half of whom are < 5 years of age. Tuberculosis in children presents unique challenges; although children are less contagious than adults, they have a higher risk of primary progression to active disease, especially infants and younger children.^3^ ^,^ ^4^ Extrapulmonary involvement and forms that are more severe, such as miliary tuberculosis and tuberculosis meningitis, are more common in pediatric tuberculosis than in adult tuberculosis.^1^

## CONTACT INVESTIGATION

Contact investigation is an evidence-based method that plays a significant role in preventing tuberculosis infection by interrupting the chain of transmission.^2^ ^,^ ^3^ The risk of developing tuberculosis is substantially higher in children who are in contact with adults and adolescents with tuberculosis. This is confirmed by the fact that the prevalence of latent tuberculosis infection (LTBI) is high among contacts.^2^ Investigation of contacts of patients with tuberculosis has been shown to increase case detection, and tuberculosis preventive treatment (TPT) can reduce the prevalence of *M. tuberculosis* transmission in the community. Assessment of people who have been exposed to tuberculosis can contribute to early diagnosis and facilitate treatment initiation, even in patients with LTBI.^2^

**Figure 1:**
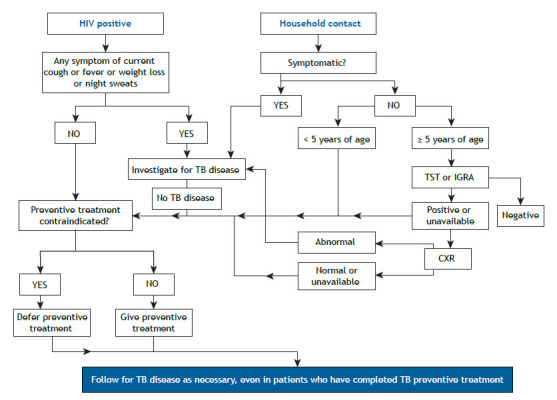
WHO algorithm for tuberculosis (TB) infection testing and TB preventive treatment in children and adolescents.^4^ TST: tuberculin skin test; IGRA: interferon gamma release assay; and CXR: chest X-ray.

The first step in contact investigation is communicating with tuberculosis patients to determine who their contacts are and inform them of the risk of developing tuberculosis.^3^ In children, combined tuberculosis screening is required, typically including symptom screening, chest X-rays, and tuberculin skin tests (TSTs) or interferon gamma release assays (IGRAs), according to the WHO (Figure 1). 

In a recently published systematic review and meta-analysis,^2^ tuberculosis prevalence among contacts was reported to be higher in low-income and high-incidence settings, a finding that shows that tuberculosis can act as a biological representation of social inequality. Because of that, contact investigation is essential not only in case detection but also in decreasing community prevalence of tuberculosis and tuberculosis mortality, especially in more vulnerable settings.^3^

## DIAGNOSIS AND TREATMENT OF LTBI IN CHILDREN

People who are infected with *M. tuberculosis* but do not have active disease are classified as having LTBI. They require TPT, which reduces the risk of progression to active tuberculosis.^1^ The diagnosis of LTBI in children can be made by a variety of methods, including the TST and IGRA. These methods are particularly important in the case of children ≤ 5 years of age with a history of contact with pulmonary tuberculosis.^4^ In Brazil, a positive (or indeterminate) IGRA result or a TST ≥ 5 mm is an indication for TPT, regardless of the time elapsed since BCG vaccination, after active tuberculosis is ruled out.^5^ According to the WHO, children < 5 years of age should be prioritized (Figure 1). Active tuberculosis should be excluded by clinical and radiological examination. 

Rifampin and isoniazid constitute the preferred TPT regimen, with daily doses of medication for three months. The recommendation is that patients receive 90 doses, ideally for three months, the number of doses being more important than the duration of the treatment. The focus should be to ensure that patients complete the full prescribed dose of medications within the specified time frame. Another option is the isoniazid-only regimen, with daily doses for six to nine months. There is evidence that 270 doses are more effective than 180 doses for patient protection.^5^

## ACTIVE TUBERCULOSIS IN CHILDREN

The diagnosis of active tuberculosis in children (< 10 years of age) is challenging because of nonspecific symptoms that are also present in common childhood infections.^1^ ^,^ ^4^ These symptoms include decreased appetite, weight loss, and chronic cough. Although active tuberculosis most commonly presents as a pulmonary disease, persistent cough (for more than three weeks) may be the only respiratory symptom, associated with progressive worsening.^5^ Although fever is not always present, it is typically above 38°C and occurs in the late afternoon. Other general signs and symptoms include anorexia, asthenia, night sweats, hepatosplenomegaly, and lymphadenopathy.^1^

Another difference between active tuberculosis in adults and active tuberculosis in children is that pulmonary tuberculosis in children is often AFB-negative, meaning that microbiological examination results are negative because of the low number of bacilli in the lesions.^5^ Therefore, the diagnosis of active tuberculosis in children is based on a combination of clinical and epidemiological criteria, associated with a nonspecific immunological test for tuberculosis infection and chest X-rays.^5^ There is no gold standard for the diagnosis of active tuberculosis in children, nor is there a universal diagnostic algorithm.^4^

The basic treatment regimen for children with active tuberculosis initially includes an intensive phase with daily doses of rifampin, isoniazid, and pyrazinamide for two months, depending on the body weight of the patient. The maintenance phase, which encompasses the next four months, includes a daily dose of rifampin and isoniazid only, also depending on the body weight of the patient.^5^ In Brazil, children and adolescents in the 3-month to 16-year age bracket with nonsevere tuberculosis, a four-month treatment course is recommended as an alternative regimen. This recommendation is based on a trial showing that a four-month treatment regimen (two months of isoniazid, rifampin, and pyrazinamide with or without ethambutol, followed by two months of isoniazid and rifampin) was noninferior to the standard six-month regimen.^6^

In conclusion, treatment outcomes for children completing tuberculosis treatment are generally excellent. Most deaths attributable to tuberculosis in children occur in those who do not receive treatment. The key to stopping the spread of tuberculosis, including children and adolescents, is to start effective tuberculosis treatment as soon as possible in people who are infected and to prevent tuberculosis disease in people at high risk of developing tuberculosis.^6^

## References

[1] Walls T, Shingadia D (2004). Global epidemiology of paediatric tuberculosis. J Infect.

[2] Velen K, Shingde RV, Ho J, Fox GJ (2021). The effectiveness of contact investigation among contacts of tuberculosis patients a systematic review and meta-analysis. Eur Respir J.

[3] Oo HS, Borry P (2024). Contact investigation in multidrug-resistant tuberculosis ethical challenges. Monash Bioeth Rev.

[4] World Health Organization [homepage on the Internet] (2022). WHO consolidated guidelines on tuberculosis: Module 5: Management of tuberculosis in children and adolescents.

[5] Brasil. Ministério da Saúde Manual de Recomendações e Controle da Tuberculose no Brasil 2a ed.

[6] World Health Organization [homepage on the Internet] (2022). WHO operational handbook on tuberculosis: module 5: Management of tuberculosis in children and adolescents.

